# Supplementary Calcium Restores Peanut (*Arachis hypogaea*) Growth and Photosynthetic Capacity Under Low Nocturnal Temperature

**DOI:** 10.3389/fpls.2019.01637

**Published:** 2020-01-21

**Authors:** Qiaobo Song, Yifei Liu, Jiayin Pang, Jean Wan Hong Yong, Yinglong Chen, Chunming Bai, Clément Gille, Qingwen Shi, Di Wu, Xiaori Han, Tianlai Li, Kadambot H. M. Siddique, Hans Lambers

**Affiliations:** ^1^ College of Land and Environment, National Key Engineering Laboratory for Efficient Utilization of Soil and Fertilizer Resources, Northeast China Plant Nutrition and Fertilization Scientific Observation and Research Station for Ministry of Agriculture and Rural Affairs, Key Laboratory of Protected Horticulture of Education Ministry and Liaoning Province, Shenyang Agricultural University, Shenyang, China; ^2^ The UWA Institute of Agriculture, The University of Western Australia, Perth, WA, Australia; ^3^ School of Agriculture and Environment, The University of Western Australia, Perth, WA, Australia; ^4^ School of Biological Sciences, The University of Western Australia, Perth, WA, Australia; ^5^ Department of Biosystems and Technology, Swedish University of Agricultural Sciences, Alnarp, Sweden; ^6^ Liaoning Academy of Agricultural Sciences, Shenyang, China; ^7^ College of Resources and Environmental Sciences, Key Laboratory of Plant-Soil Interactions, Ministry of Education, National Academy of Agriculture Green Development, China Agricultural University, Beijing, China

**Keywords:** peanut, low nocturnal temperature, growth, calcium, photosynthesis

## Abstract

Peanut (*Arachis hypogaea* L.) is a globally important oil crop, which often experiences poor growth and seedling necrosis under low nocturnal temperatures (LNT). This study assessed the effects of supplementary calcium (Ca^2+^) and a calmodulin inhibitor on peanut growth and photosynthetic characteristics of plants exposed to LNT, followed by recovery at a higher temperature. We monitored key growth and photosynthetic parameters in a climate-controlled chamber in pots containing soil. LNT reduced peanut growth and dry matter accumulation, enhanced leaf nonstructural carbohydrates concentrations and non-photochemical quenching, decreased the electron transport rate, increased the transmembrane proton gradient, and decreased gas exchange rates. In peanuts subjected to LNT, foliar application of Ca^2+^ restored growth, dry matter production and leaf photosynthetic capacity. In particular, the foliar Ca^2+^ application restored temperature-dependent photosynthesis feedback inhibition due to improved growth/sink demand. Foliar sprays of a calmodulin inhibitor further deteriorated the effects of LNT which validated the protective role of Ca^2+^ in facilitating LNT tolerance of peanuts.

## Introduction

*Arachis hypogaea* L. (peanut or groundnut), originally from tropical South America (Bolivia and adjoining countries), is primarily grown in tropical and subtropical agro-climatic areas of Asia, Africa, Oceania, and the Americas. It is an important oil crop globally, providing the main source of edible oil and protein in many developing countries (Prasad et al., 2003; Bertioli et al., 2016). Low-temperature stress, particularly low nocturnal temperature (LNT), is a major limiting factor curtailing productivity and limiting the cultivation distribution of peanuts (Wan, 2003). Tropical and subtropical plants are generally sensitive to chilling stress due to a lack of cold acclimation (Zhu et al., 2007; Liu et al., 2013; Hajihashemi et al., 2018). Low-temperature stress often negatively influences plant growth, development and photosynthetic carbon assimilation, especially during early growth. Low-temperature stress significantly reduced leaf area in rice (Zhou et al., 2018), maize (Wang et al., 2018), sunflower, sorghum (Tardieu et al., 1999) and Chinese crab apple seedlings (Li et al., 2017) and inhibited root growth and dry matter accumulation in maize (Mozafar and Oertli, 1990; Wang et al., 2018). In addition, low-temperature stress reduced the tillering rate and leaf expansion in rice (Huang et al., 2013; Liu et al., 2018) and induced rice spikelet sterility (Haque, 1988). The frequent and intense extreme climate environments of LNT stress followed by warm sunny days are common in temperate peanut-cultivating regions globally, particularly in north China (Wan, 2003). Peanut often experiences poor growth and seedling necrosis under LNT stress, which severely reduces peanut yield and seed quality (Bagnall et al., 1988; Wan, 2003; Liu et al., 2013).

Plants of tropical or subtropical origin are often susceptible to suboptimal, but non-freezing (chilling) temperature environments (Bauer et al., 1985; Damian and Donald, 2001; Zhu et al., 2007; Liu et al., 2013). LNT stress significantly reduces leaf growth and Chl a and Chl b concentrations in grapevine, which has a negative impact on photosynthesis (Bertamini et al., 2005). Photosynthesis is very sensitive to LNT stress (Allen et al., 2000; Yu et al., 2002; Zhang et al., 2014; Hajihashemi et al., 2018). LNT stress inhibits carbon fixation reactions and photosystem II (PSII) repair by suppressing *de novo* synthesis of the D1 protein and photoreaction center activity (Allakhverdiev and Murata, 2004; Murata et al., 2007; Liu et al., 2012). Temperature changes have a strong impact on photosynthetic reactions. When air temperature declined by 10°C, the activity of enzymes associated with carbon assimilation reduced by 50% (Yamori, 2016). The reduced consumption of NADPH results in the subsequent accumulation of reductants downstream of photosystem I (PSI) (Donald et al., 1996; Yamori and Shikanai, 2016). Furthermore, both PSI and PSII accelerate the production of reactive oxygen species under excess excitation energy which causes photoinhibition (Asada, 2006). Plants have a highly responsive regulatory system to prevent photodamage when subjected to chilling stress (Yamori, 2016). In addition to harnessing a non-photochemical quenching (NPQ) mechanism, which serves to dissipate excess excitation energy accumulated in PSII without causing adverse effects; cyclic electron flow (CEF) is another major photoprotection mechanism (Bukhov et al., 1999; Zhang et al., 2014).

Calcium, an essential element for plants, serves not only as a structural component in plant cells but also as a key signaling molecule involved in multiple signal-transduction pathways in its ionic form Ca^2+^ (White and Broadley, 2003; Tian et al., 2019). In particular, calcium has well-documented roles in mediating plant responses to abiotic and biotic stimuli (Cachorro et al., 1994; Skórzyńska-Polit et al., 1998; White and Broadley, 2003; Ding et al., 2018; He et al., 2018; Naeem et al., 2018; Patrick et al., 2018). Low-temperature stress often leads to an increase in free Ca^2+^ in plants, followed by cold-induced protein phosphorylation and the accumulation of the cold acclimation-specific genes that improve the adaptation of plants to cold stress (Monroy and Dhindsa, 1995). In addition, exogenous calcium improves the cold tolerance of plants through two ways: one is the maintenance of the cell membrane and cell wall structure, and an enhanced activity of protective enzymes; the other is the transfer of low-temperature signals which induce the expression of cold-tolerance genes (White and Broadley, 2003; Li et al., 2017). In response to chilling stress, the pre-treatment of exogenous Ca^2+^ significantly improved the physiological response including growth and photosynthesis in low-temperature sensitive plant species such as peanut (Liu et al., 2013), wheat (You et al., 2002), Chinese crab apple (Li et al., 2017) and tomato (Zhang et al., 2014). It is generally believed that plant cell wall, mitochondria and chloroplasts have enormous capacity to store Ca^2+^ (Hepler, 2005; Chen et al., 2015); moderate Ca^2+^ concentrations can sustain cell wall growth and membrane integrity as well as osmotic functioning (Poovaiah and Leopold, 1973; Hepler, 2005; Qu et al., 2012); mitochondrial NADH dehydrogenase activity is regulated by Ca^2+^ (Anderson and Cormier, 1978; Møller et al., 1981); furthermore, the Ca^2+^/calmodulin is involved in the regulation of NAD kinase and photosynthesis (Jarrett et al., 1982; Rocha and Vothknecht, 2012). In our previous study, exogenous Ca^2+^ enhanced peanut photosynthate production under LNT stress (Liu et al., 2013); however, its underlying physiological mechanisms are largely unknown. Therefore, this study examined the effects of exogenous Ca^2+^ and a calmodulin inhibitor, trifluoperazine (TFP, which have been extensively used to demonstrate calmodulin-mediated plant growth response), on the growth and photosynthetic function of peanut exposed to LNT and the following recovery from LNT stress.

## Materials and Methods

### Plant Material and Experimental Design

Fenghua 1, the common high-yielding peanut cultivar in China, was used in this study. Uniform seeds of peanut were pre-germinated in a Petri dish for one day at 27°C and then planted in 32-cavity trays for 7 days before selecting 72 uniform peanut seedlings for transplantation into 72 pots (200 mm height, 260 mm diameter) filled with 4 kg of standard horticultural nutrient substrate (Changchun Xihe Agro-technology co. Ltd, Jilin, China). The pots were then placed in an artificial climate chamber (Conviron, Winnipeg, Canada), with a daytime temperature of 25°C and nocturnal temperature of 20°C at a relative humidity (RH) of 60 ± 5%. All plants received a 12 h daytime photoperiod at a photosynthetic photon flux density (PPFD) of 600 μmol quanta·m^–2^·s^–1^ and a CO_2_ concentration of 400 ± 5 μmol·mol^–1^. After 3 days of acclimation, the pots were divided equally into four treatment groups: (1) CK (normal nocturnal temperature of 20°C/normal daytime temperature of 25°C + foliar spray of type 1 ultrapure water), (2) LNT (LNT of 10°C/normal daytime temperature of 25°C + foliar spray of type 1 ultrapure water), (3) LNT + Ca (LNT of 10°C/normal daytime temperature of 25°C + foliar spray of 15 mmol·L^–1^ CaCl_2_) and (4) LNT + TFP (LNT of 10°C/normal daytime temperature of 25°C + foliar spray of 5 mmol·L^–1^ TFP). CK was defined as the normal temperature control group at normal nocturnal temperature (20°C), while the other groups were subjected to LNT (10°C) stress for 5 days (5 DoT, days of LNT treatment). After 5 days of LNT stress, the peanut seedlings from (2), (3), and (4) treatment groups mentioned above were recovered for another 5 days (5 DoR, days of normal temperature recovery) at a normal nocturnal temperature of 20°C and a normal daytime temperature of 25°C to simulate the common cold wave with LNT attack and recession in peanut production.

The optimal levels of exogenous Ca^2+^ (15 mmol·L^–1^ CaCl_2_) and TFP (5 mmol·L^–1^ TFP), a calmodulin (CaM) inhibitor which disrupts the binding between Ca^2+^/calmodulin and effector proteins, as well as the application technique, were established in a previous experiment (Liu et al., 2013). Leaves were sprayed carefully and evenly using moisture sprayers 3 days before the LNT stress (twice a day for 3 days). The LNT treatments were subjected to 12 h (from 18:00 to 6:00 h) LNT treatments at 10°C by transferring the peanut seedlings to an artificial climate chamber (Conviron, Winnipeg, Canada).

### Plant Sampling and Measurements

Three peanut seedlings per treatment were sampled twice—after 5 days of LNT treatment (5 DoT, days of LNT treatment) and after another 5 days of recovery from LNT stress (5 DoR, days of normal temperature recovery) for the measurement of leaf area and photosynthetic pigments. Leaf area was measured using an LI-3000C (LI-COR Biosciences, Lincoln NE, USA). Chlorophyll a, chlorophyll b and carotenoid concentrations of the third youngest fully expanded leaves were determined using the spectrophotometer method (Lichtenthaler, 1987). Another six peanut seedlings from each treatment were sampled at 5 DoT and 5 DoR. All samples were oven-dried at 105°C for 30 min and then at 70°C to a constant weight. In addition, oven-dried leaflets from the third youngest fully expanded leaves of six peanut seedlings per treatment were pooled in three biological replicates and ground to a powder, and a total of three biological replicates/pools (six peanut seedlings) were used for carbohydrates measurements. Soluble sugars were extracted from approximately 100 mg of the oven-dried leaf powder with 80% (v/v) ethanol at 85°C and quantified using the microtiter method (Hendrix, 1993). Pellets containing starch were oven-dried overnight at 60°C. Starch in the pellet was first gelatinized by addition of 1 ml of 0.2 mol·l^–1^ KOH and incubated in a boiling water bath for 30 min (Rufty and Huber, 1983). After cooling, 0.2 ml of 1 mol·l^–1^ acetic acid was added, and the solution incubated with 2 ml acetate buffer (pH 4.6) containing amyloglucosidase (six units, Roche) at 55°C for 1 h. The reaction was terminated in a boiling water bath, and the resulting supernatant analyzed for glucose.

Leaf gas exchange was measured on the third youngest fully expanded leaves using an open system of gas exchange equipment (GFS-3000, Heinz Walz GmbH, Effeltrich, Germany) at 1, 3, and 5 DoT and 1, 3, and 5 DoR. During gas exchange measurements, the leaf cuvette temperature was set to 25°C and 60% RH. The CO_2_ concentration was maintained at 400 μmol·mol^–1^. An LED array provided a PPFD of 600 μmol quanta·m^–2^·s^–1^. The third youngest fully expanded leaf was kept in the chamber by ensuring the thermocouple touching it from the underside. Gas exchange parameters included net photosynthetic rate (Pn), stomatal conductance (g_s_), atmospheric CO_2_ concentration (C_a_), transpiration rate (Tr), intercellular CO_2_ concentration (C_i_), water-use efficiency (WUE = Pn/Tr), and leaf stomatal limitation *(Ls* = 1 – *C_i_*/*C_a_*).

The software Dual-PAM v1.19 was used to control the Dual-PAM 100 measuring system (Heinz Walz, Effeltrich, Germany) and measure chlorophyll fluorescence and P700 parameters on the third youngest fully expanded leaf (*ca*. 1 cm^2^); all steps were carried out in accordance with the standard protocols provided by the manufacturer (Heinz Walz, Effeltrich, Germany) and earlier research (Shi et al., 2019). The fluorescence slow-kinetics were measured after a dark adaptation of 30 min. The intensity of saturation pulse light (red light) and actinic light (red light) were set as 10,000 and 132 μmol quanta·m^–2^·s^–1^, respectively. Chlorophyll fluorescence parameters were calculated as follows: Fo and Fm are the minimum and maximum fluorescence yields of the dark-adjusted sample with all PSII center open and closed, respectively. Fo’ and Fm’ are the minimum and maximum fluorescence yield of the illuminated sample with some PSII center open and closed, respectively. F is the fluorescence yields measured briefly before applying a saturation pulse. Fv/Fm = (Fm – Fo)/Fm indicates the maximal/intrinsic photochemical efficiency of PSII (Kitajima and Butler, 1975). Y(II) = (Fmʹ– F)/Fm is the actual quantum yield of PSII (Genty, 1989). Y(NO) = F/Fm is the non-regulated energy loss in PSII. Y(NO) represents the fraction of energy that is dissipated as heat and fluorescence, and high values of Y(NO) reflects the inability of the plant to protect itself against damage by excess excitation (Cailly et al., 1996; Klughammer and Schreiber, 2008). Y(NPQ) = 1 – Y(II) – Y(NO) is the regulatory quantum yield in PSII and represents the fraction of energy dissipated in the form of heat through the regulated photoprotective NPQ mechanism (Kramer et al., 2004). ETR(II) = PAR·Y(II)·0.84·0.5 is the relative electron transfer rate in PSII. PAR (μmol quanta·m^–2^·s^–1^) is photosynthetically active radiation (Genty, 1989; Schreiber et al., 1995).

The PSI photosynthetic parameters were measured using a Dual-PAM 100 device based on the P700 signal (absorption differences between 830 and 875 nm). The quantum yields of PSI were determined using the saturation pulse method (Klughammer and Schreiber, 1994). The P700 parameters were calculated as follows: Y(NA) = (Pm – Pmʹ)/Pm is the quantum yield of PSI non-photochemical energy dissipation due to the acceptor-side limitation. Y(ND) = 1 – *P*700*_red_* is the quantum yield of PSI non-photochemical energy dissipation due to the donor-side limitation (Cailly et al., 1996). Y(I) = 1 – Y(NA) – Y(ND) is the actual quantum yield in PSI under light (Klughammer and Schreiber, 1994; Klughammer and Schreiber, 2008). ETR(I) = PAR·Y(I)·0.84·0.5 is the relative electron transfer rate in PSI (Klughammer and Schreiber, 2008). Pm is the maximum oxidation state of PSI under far-red light (720 nm). Pm’ is the maximum oxidation state of PSI under actinic light (420 nm). P700_red_ is the P700 reduction parameter under the light. The CEF was estimated as CEF = ETR(I) – ETR(II). Similarly, Y(CEF)/Y(II) = [Y(I) – Y(II)]/Y(II) was calculated as the ratio of the quantum yield of CEF to Y(II) and later used to estimate cyclic electron transfer (Munekage et al., 2002; Yamori and Shikanai, 2016).

The dual-beam 550 nm to 515 nm difference signal (electrochromic shift) was monitored simultaneously using the P515/535 module of the Dual-PAM 100 (Klughammer and Schreiber, 2008; Zhang et al., 2014). Three independent peanut seedlings per treatment were selected at 5 DoT for the determination of the following indicators. After 1 h of dark acclimation, P515 changes induced by saturating single turnover flashes were recorded to evaluate thylakoid membrane integrity. After 10 min of pre-illumination at 600 μmol quanta·m^–2^·s^–1^ and 4 min of dark acclimation, P515 changes induced by saturating single turnover flashes were recorded to evaluate ATP-synthase activity. Slow dark–light–dark induction transients of the 550 to 515 nm signals reflected changes in membrane potential (electrochromic pigment absorbance shift). Actinic light (AL; 600 μmol quanta·m^−2^·s^−1^) was turned on at 30 s and off at 330 s.

### Statistical Analysis

Statistical analyses were carried out using one-way ANOVA in SPSS 19.0. A total of 72 uniform peanut seedlings were included in this study, which were allocated to four different treatments (i.e. 18 seedlings per treatment). Three out of the 18 peanut seedlings per treatment were used for the non-destructive measurement of leaf gas exchange, chlorophyll fluorescence and P700 parameters. The remaining peanut seedlings per treatment were used for the destructive sampling for peanut seedlings growth observation and photography as well as the measurement of leaf area, biomass accumulation, photosynthetic pigments concentrations, and carbohydrates. The results were presented as mean values and standard error of three biological replicates. Post-hoc LSD tests at *P* = 0.05 were performed to highlight the differences among the four treatments. The significant differences *P*-value is indicated as the *(*P* ≤ 0.05) and **(*P* ≤ 0.01), respectively, among the treatments.

## Results

### Effect of Exogenous Calcium (Ca^2+^) and a Calmodulin Inhibitor (TFP) on Peanut Growth After 5 Days of Low Nocturnal Temperature Stress Followed by 5 Days of Recovery

**Figure 1:**
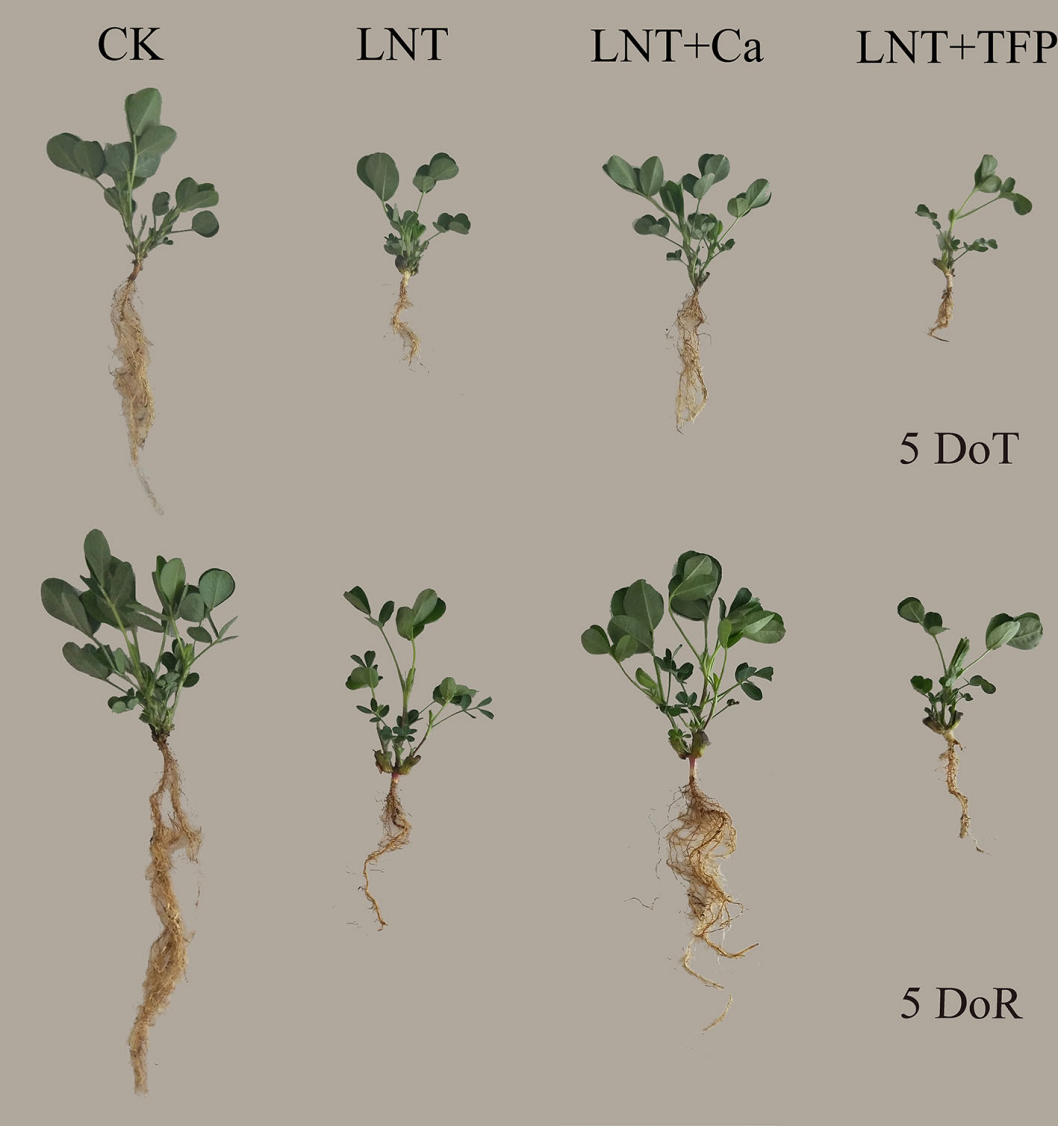
Effect of exogenous calcium (Ca^2+^) and a calmodulin inhibitor (TFP) on peanut growth after 5 days of low nocturnal temperature (LNT) (5 DoT) followed by 5 days of recovery (5 DoR). CK, normal nocturnal temperature of 20°C/normal daytime temperature of 25°C + foliar spray of type 1 ultrapure water; LNT, low nocturnal temperature of 10°C/normal daytime temperature of 25°C + foliar spray of type 1 ultrapure water; LNT + Ca, low nocturnal temperature of 10°C/normal daytime temperature of 25°C + foliar spray of 15 mmol·l^–1^ CaCl_2_; LNT + TFP, low nocturnal temperature of 10°C/normal daytime temperature of 25°C + foliar spray of 5 mmol·l^–1^ TFP.

The LNT treatment significantly inhibited peanut growth, which did not recover after 5 days of recovery (**Figure 1**). Exogenous Ca^2+^ application (LNT + Ca) counteracted the LNT stress and benefited the recovery process. The calmodulin inhibitor (LNT + TFP) further reduced peanut growth and biomass when compared with LNT (**Figure 1**).

The LNT treatment significantly decreased leaf area in peanut. The LNT + Ca treatment had more leaf area than the LNT treatment, while the LNT + TFP treatment had less. LNT stress reduced dry matter accumulation in the roots, stems and leaves. The LNT + Ca treatment significantly increased root, stem and leaf dry weights, relative to LNT. The control (CK) had the highest chlorophyll a and b concentrations, followed by LNT + Ca and LNT, with the lowest in LNT + TFP. LNT stress significantly reduced the chlorophyll a and b concentrations while had no significant effect on the carotenoid level. LNT + Ca significantly enhanced the chlorophyll a and b concentrations in peanuts leaves. No significant difference in carotenoid concentration was observed among the four treatments at either 5 DoT or 5 DoR (**Table 1**).

**Table 1:** Effect of exogenous calcium (Ca^2+)^ and a calmodulin inhibitor (TFP) on peanut leaf area, dry matter and pigment concentrations (chlorophyll a and b, carotenoid) after 5 days low nocturnal temperature (LNT) (5 DoT) followed by 5 days of recovery (5 DoR).

Time	Treatments	Leaf area (cm^2^)	Dry weight (mg) **Root**			Chlorophyll a concentration (mg·g^–1^)	Chlorophyll b concentration (mg·g^–1^)	Carotenoid concentration(mg·g^–1^)
			Root	Stem	Leaf			
5 DoT	CK	52.7 ± 0.4a	215 ± 1a	527 ± 7a	480 ± 6a	1.56 ± 0.02a	0.81 ± 0.02a	0.24 ± 0.01a
	LNT	38.9 ± 0.8c	87 ± 3c	309 ± 8c	345 ± 5c	1.38 ± 0.01c	0.66 ± 0.01c	0.27 ± 0.02a
	LNT + Ca	46.5 ± 0.4b	147 ± 2b	401 ± 7b	410 ± 7b	1.47 ± 0.01b	0.73 ± 0.01b	0.26 ± 0.01a
	LNT + TFP	32.8 ± 0.2d	70 ± 3d	256 ± 7d	284 ± 8d	1.33 ± 0.01d	0.61 ± 0.02d	0.25 ± 0.01a
5 DoR	CK	79.8 ± 0.3a	360 ± 6a	932 ± 15a	784 ± 15a	1.79 ± 0.03a	0.93 ± 0.02a	0.28 ± 0.01a
	LNT	53.5 ± 0.8c	161 ± 3c	761 ± 10c	569 ± 15c	1.45 ± 0.01b	0.74 ± 0.02c	0.32 ± 0.01a
	LNT + Ca	65.7 ± 1.5b	207 ± 3b	836 ± 17b	644 ± 21b	1.72 ± 0.05a	0.87 ± 0.01b	0.29 ± 0.01a
	LNT + TFP	48.3 ± 1.3d	132 ± 1d	751 ± 8c	441 ± 20d	1.41 ± 0.01b	0.67 ± 0.01d	0.29 ± 0.02a

Values are means of three biological replicates ± SE (n = 3). Different letters (a, b, c, d) in the same column indicate significant differences among the treatments by LSD test (P ≤ 0.05).

### Effect of Exogenous Calcium (Ca^2+^) and a Calmodulin Inhibitor (TFP) on Solub rations of Peanut Leaves After 5 Days of Low Nocturnal Temperature Stress Followed by 5 Days of Recovery

**Figure 2:**
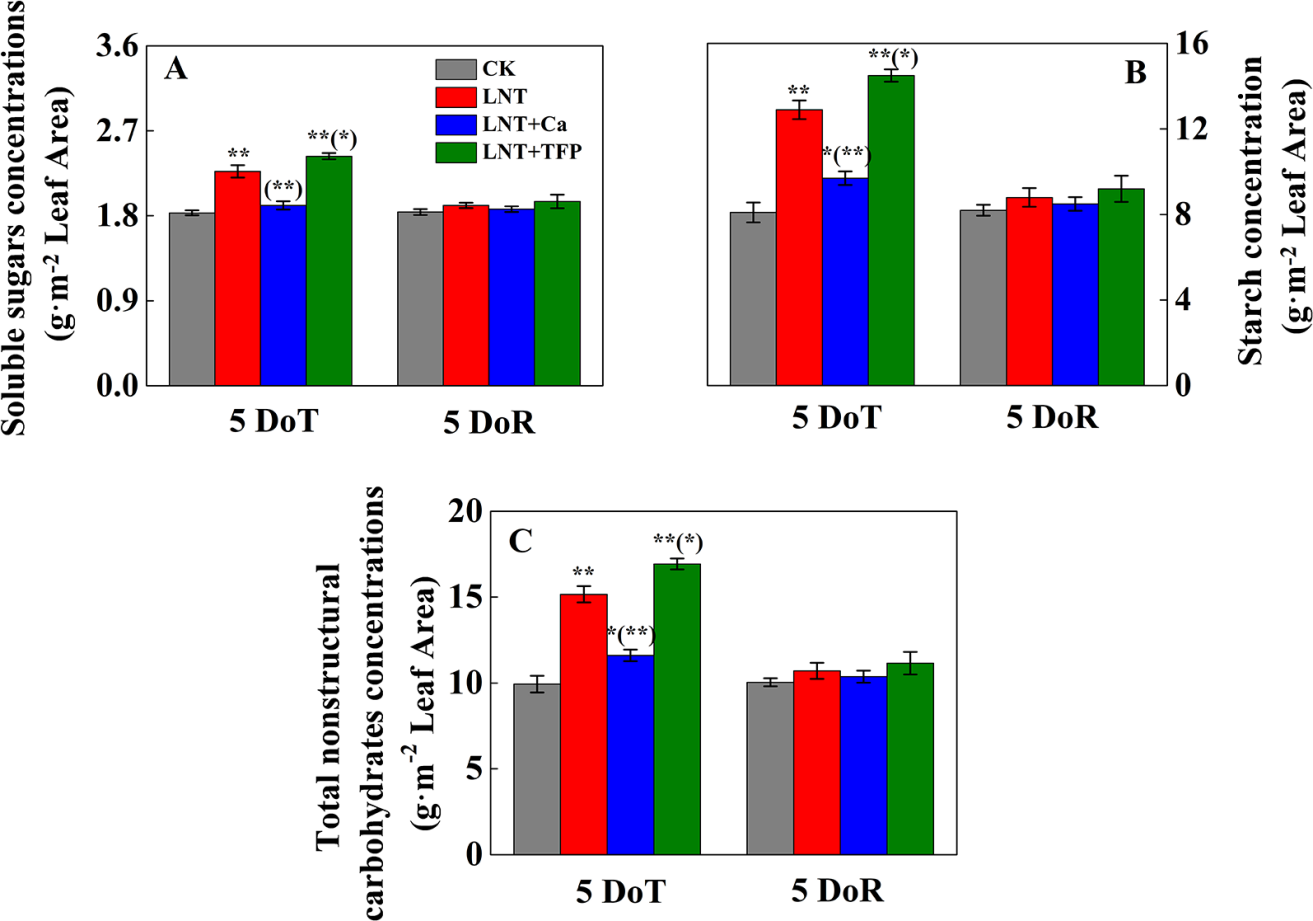
Effect of exogenous calcium (Ca^2+^) and a calmodulin inhibitor (TFP) on the concentration of soluble sugars **(A)**, starch **(B)** and total nonstructural carbohydrates **(C)** after 5 days of low nocturnal temperature (LNT) (5 DoT) followed by 5 days of recovery (5 DoR). Values are means of three biological replicates ± SE (*n* = 3). * and ** indicate significant differences at *P* ≤ 0.05, and *P* ≤ 0.01, respectively, among the treatments. The significance between the three treatments under LNT and CK at 5 DoT and 5 DoR was shown in parenthesis.

The LNT treatment significantly increased the concentration of soluble sugars in leaves at 5 DoT (**Figure 2A**); the LNT + Ca treatment produced lower and the LNT + TFP treatment produced higher concentrations of soluble sugars than the LNT treatment. The CK treatment had the lowest concentrations of starch and total nonstructural carbohydrates at 5 DoT, followed by LNT + Ca and LNT, and LNT + TFP with the highest (**Figures 2B, C**). Soluble sugar, starch and total nonstructural carbohydrate concentrations did not differ between treatments at 5 DoR.

### Effect of Exogenous Calcium (Ca^2+^) and a Calmodulin Inhibitor (TFP) on Gas Exchange During 5 Days Low Nocturnal Temperature Stress Followed by 5 Days of Recovery

**Figure 3:**
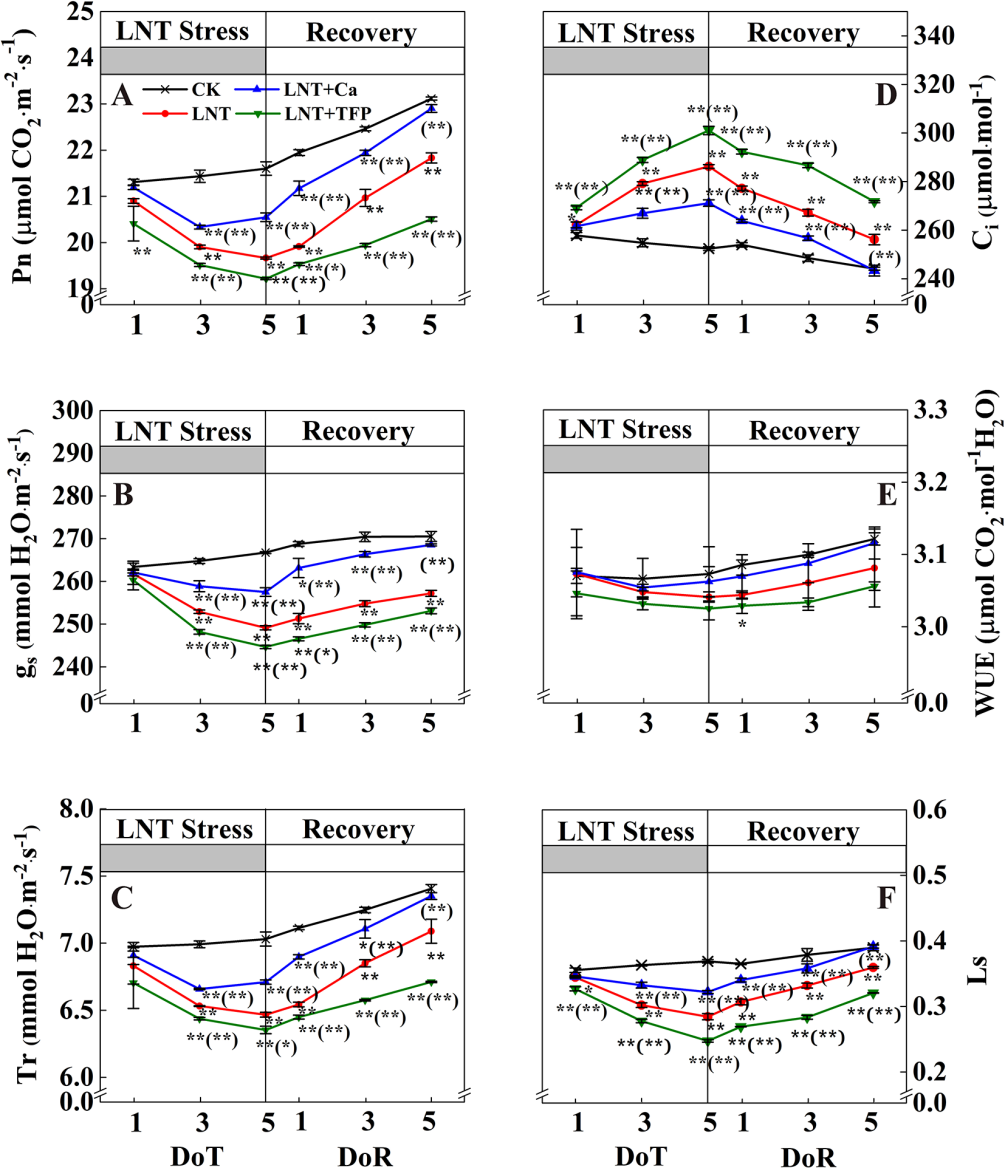
Effect of exogenous calcium (Ca^2+^) and a calmodulin inhibitor (TFP) on peanut gas exchange characteristics [net photosynthetic rate (Pn, **A**), stomatal conductance (g_s_, **B**), transpiration rate (Tr, **C**), intercellular CO_2_ concentration (C_i_, **D**), water-use efficiency (WUE, **E**) and leaf stomatal limitation (Ls, **F**)] during 5 days of low nocturnal temperature (1, 3, and 5 DoT) followed by 5 days of recovery (1, 3 and 5 DoR). Values are means of three biological replicates ± SE (*n* = 3). * and ** indicate significant differences at *P* ≤ 0.05, and *P* ≤ 0.01, respectively, among the treatments. The significance between the three treatments under LNT and CK was shown in parenthesis.

Leaf gas exchange parameters did not differ between treatments at 1 DoT, but CK and LNT differed at 3 and 5 DoT. The LNT treatment reduced Pn, g_s_, Tr, and Ls (**Figure 3**) and increased C_i_ significantly. Compared with LNT, LNT + Ca significantly increased Pn, g_s_, Tr, and Ls and decreased C_i_ while LNT + TFP significantly reduced Pn, g_s,_ Tr, and Ls, but increased C_i_ further. During the 5 days recovery at a normal nocturnal temperature, LNT-treated plants continued to increase Pn, g_s,_ Tr, and Ls and decrease C_i_. The same pattern was observed for LNT + Ca but at a slightly lower level.

### Effect of Exogenous Calcium (Ca^2+^) and a Calmodulin Inhibitor (TFP) on Peanut Photosystem Activities During 5 Days of Low Nocturnal Temperature Stress Followed by 5 Days of Recovery

**Figure 4:**
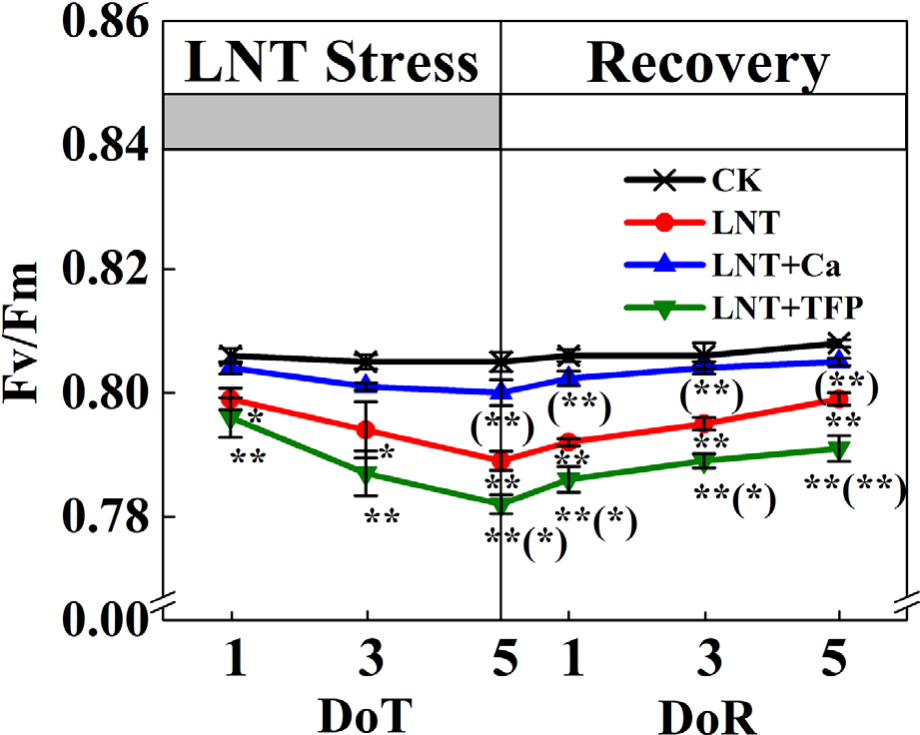
Effect of exogenous calcium (Ca^2+^) and a calmodulin inhibitor (TFP) on peanut maximum quantum yield of PSII (Fv/Fm) during 5 days of low nocturnal temperature (LNT) stress followed by 5 days of recovery. Values are means of three biological replicates ± SE (*n* = 3). * and ** indicate significant differences at *P* ≤ 0.05, and *P* ≤ 0.01, respectively, among the treatments. The significance between the three treatments under LNT and CK was shown in parenthesis.

The maximum quantum yield of PSII (Fv/Fm) declined markedly under LNT stress (**Figure 4**). In particular, the Fv/Fm of the LNT + Ca treatment recovered fully by 5 DoR, it was significantly higher than that of LNT and LNT + TFP.

**Figure 5:**
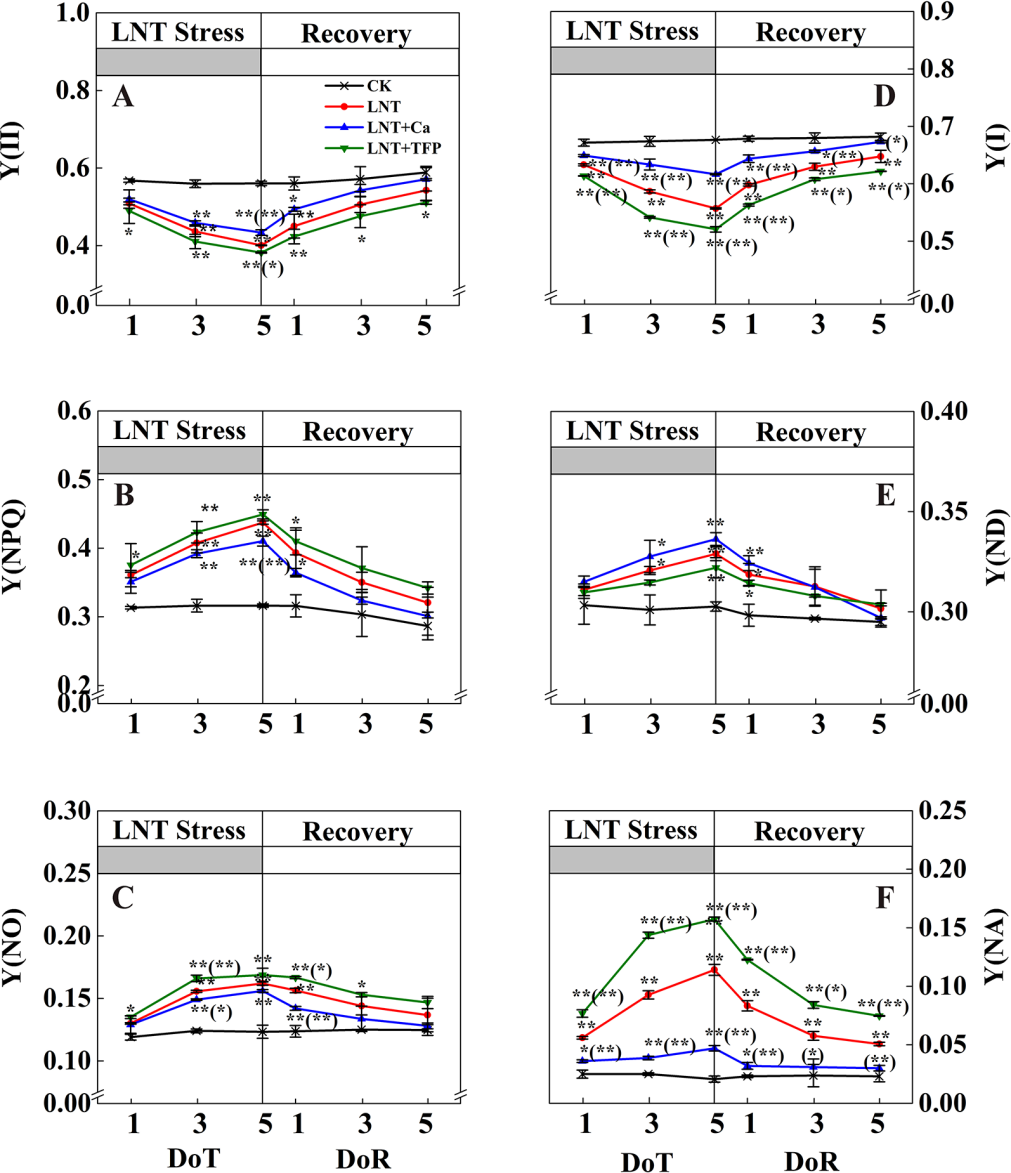
Effect of exogenous calcium (Ca^2+^) and a calmodulin inhibitor (TFP) on peanut photosystems parameters during 5 days of low nocturnal temperature (LNT) stress followed by 5 days of recovery. **(A)** Y(II): PSII photochemistry effective quantum yield; **(B)** Y(NPQ): PSII regulated energy dissipation quantum yield; **(C)** Y(NO): PSII non-regulated energy dissipation quantum yield; **(D)** Y(I): PSI photochemistry effective quantum yield; **(E)** Y(ND): PSI non-photochemical energy dissipation due to the donor-side limitation; **(F)** Y(NA): PSI non-photochemical energy dissipation due to the acceptor-side limitation. Values are means of three biological replicates ± SE (*n* = 3). * and ** indicate significant differences at *P* ≤ 0.05, and *P* ≤ 0.01, respectively, among the treatments. The significance between the three treatments under LNT and CK was shown in parenthesis.

The LNT treatment decreased Y(II) significantly (**Figure 5A**). The actual quantum yield of PSII in LNT treatments declined significantly and dissipated excess energy by gradually increasing Y(NPQ), the regulatory quantum yield of PSII (**Figure 5B**). Y(II) tended to decrease gradually with the onset of LNT stress. The heat dissipation was not enough to dissipate the excess excitation energy in LNT treatments. Consequently, Y(NO) increased to a higher level (**Figure 5C**). During the 5 days recovery at a normal nocturnal temperature, Y(II) recovered gradually, while Y(NO) and Y(NPQ) decreased slowly. In general, the PSII self-repair process in the LNT + Ca treatment was significantly greater than that in the LNT and LNT + TFP treatments. LNT stress decreased Y(I) and increased Y(NA) (**Figures 5D, F**). At 1 DoT, Y(ND) did not differ between treatments (**Figure 5E**), increasing gradually during the LNT stress; by 5 DoT, the LNT + Ca treatment had higher Y(ND) than LNT and LNT + TFP (**Figure 5E**). At 5 DoT, the LNT + Ca treatment had significantly higher Y(I) and lower Y(NA) than LNT, while the LNT + TFP treatment had significantly higher Y(NA) and lower Y(I) than LNT. During the recovery, Y(ND) did not differ between treatments. It is noteworthy that Y(NA) and Y(I) in the LNT and LNT + TFP treatments were partially restored during the recovery, but not to the same levels as those in LNT + Ca and CK (**Figures 5D, F**).

**Figure 6:**
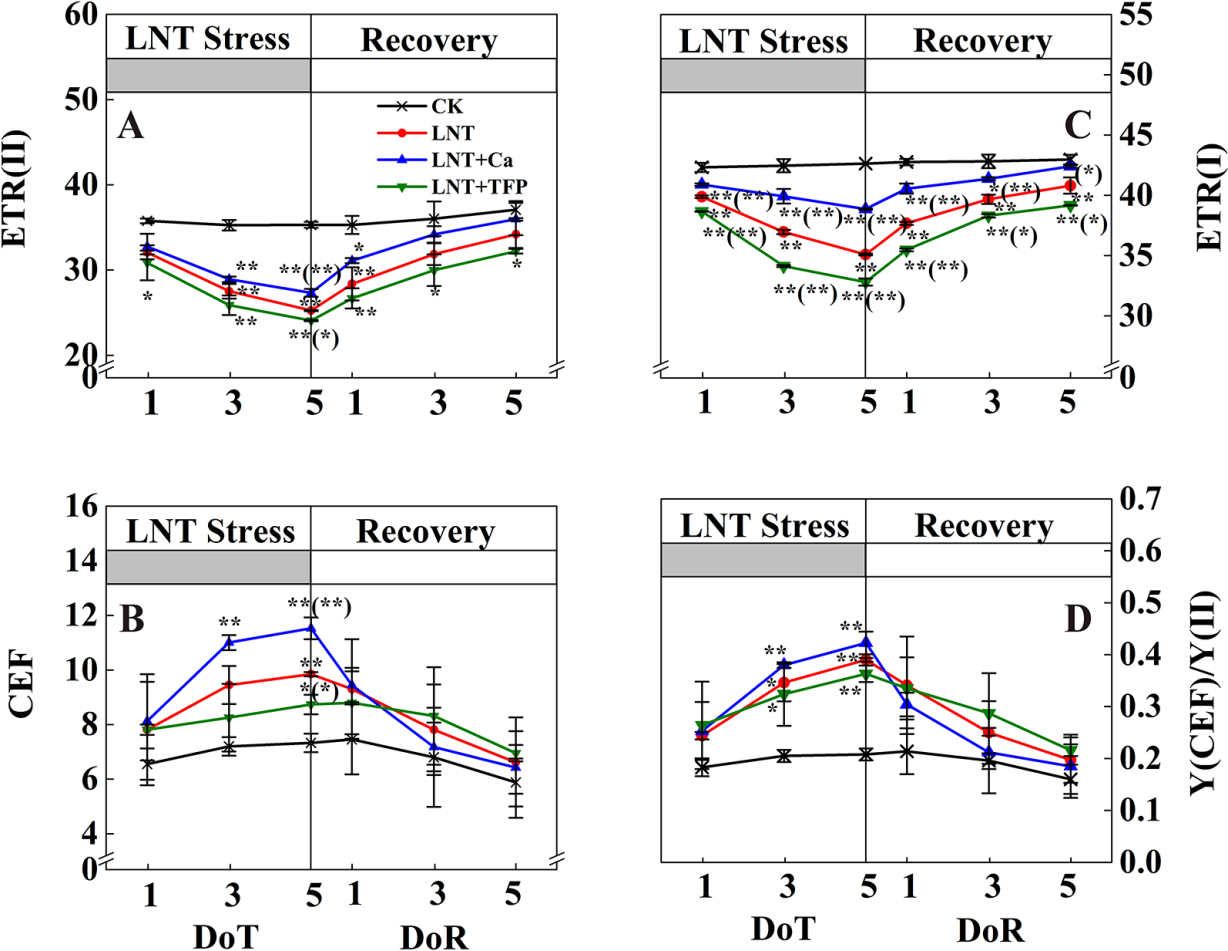
Effect of exogenous calcium (Ca^2+^) and a calmodulin inhibitor (TFP) on peanut photosynthetic electron transport during 5 days of low nocturnal temperature (LNT) stress followed by 5 days of recovery. **(A)** ETR(II): PSII photosynthetic electron transport rate; **(B)** CEF: cyclic electron flow around PSI; **(C)** ETR(I): PSI photosynthetic electron transport rate; **(D)** Y(CEF)/Y(II): the ratio of quantum yield of CEF to Y(II). Values are means of three biological replicates ± SE (*n* = 3). * and ** indicate significant differences at *P* ≤ 0.05, and *P* ≤ 0.01, respectively, among the treatments. The significance between the three treatments under LNT and CK was shown in parenthesis.

The LNT treatment reduced ETR(II) and ETR(I) and enhanced CEF and Y(CEF)/Y(II) (**Figure 6**). Compared with LNT, LNT + Ca had higher ETR(II), ETR(I), CEF and Y(CEF)/Y(II), while LNT + TFP had lower ETR(II), ETR(I), CEF and Y(CEF)/Y(II). During the recovery, ETR(II) and ETR(I) of LNT and LNT + Ca treatments increased rapidly, while CEF and Y(CEF)/Y(II) decreased, more so in LNT + Ca. At 5 DoR, ETR(II) and ETR(I) of LNT + TFP had not recovered to the control level and were significantly lower than those of the other treatments.

### Effect of Exogenous Calcium (Ca^2+^) and a Calmodulin Inhibitor (TFP) on the Proton Motive Force, Thylakoid Membrane Integrity and ATP-Synthase Activity of Peanut Leaves After Low Nocturnal Temperature Stress

**Figure 7:**
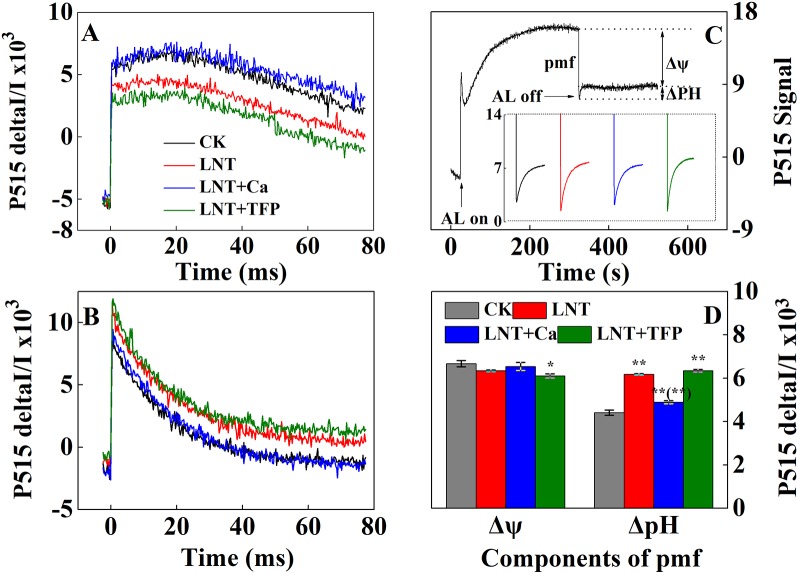
Effect of exogenous calcium (Ca^2+^) and a calmodulin inhibitor (TFP) on thylakoid membrane activity in peanut leaves after 5 days of low nocturnal temperature (LNT) stress. **(A)** Rapid kinetics of P515 induced by saturating single turnover flashes in peanut leaves after dark acclimation for 1 h; **(B)** Fast kinetics of P515 induced by saturating single turnover flashes in peanut leaves after pre-illumination for 10 min at 600 μmol photons·m^−2^·s^−1^ followed by 4 min darkness; **(C)** Proton gradient (△pH) and **(D)** membrane potential (△ψ) by using the slow ‘dark–light–dark’ signal induction transients of 515 nm signal after fully dark-acclimated (12 h). Values are means of three biological replicates ± SE (*n* = 3). * and ** indicate significant differences at *P* ≤ 0.05, and *P* ≤ 0.01, respectively, among the treatments. The significance between the three treatments under LNT and CK at 5 DoT was shown in parenthesis.

The LNT treatment significantly reduced thylakoid membrane integrity and ATPase activity, based on P515 signals (**Figures 7A, B**). The LNT + Ca treatment maintained thylakoid membrane integrity and ATPase activity in peanut leaves, while LNT + TFP further exacerbated thylakoid membrane damage. The LNT treatment also reduced thylakoid membrane potential (Δψ) and increased transmembrane proton potential (ΔpH) (**Figures 7C, D**). Compared with LNT, LNT + Ca increased Δψ and decreased ΔpH markedly, while LNT + TFP had the opposite effect.

## Discussion

### Effect of Low Nocturnal Temperature on Peanut Growth and Photosynthetic Capacity

We demonstrated that LNT stress significantly inhibited peanut growth, which could not be fully restored during the recovery period (**Figure 1**). Previous studies also showed that, below the peanut threshold temperature of 15°C, the leaves usually exhibit poor growth and necrotic injury in the field (Wan, 2003). Exposing peanuts to a dark chilling environment significantly inhibits seedling growth, with reduced leaf area and shoot and root dry matter accumulation (Solanke and Sharma, 2008; Dias et al., 2011; Hajihashemi et al., 2018). While leaf expansion rates generally vary with air temperature, low-temperature stress can reduce the rates of leaf initiation and expansion and final leaf area in sunflower, maize and sorghum (Tardieu et al., 1999). LNT stress also reduced leaf growth, the concentration of photosynthetic pigments and the shoot and root dry matter accumulation in tomato (Latef and He, 2011; Liu et al., 2011), and damaged leaf structure and growth in tobacco (Kasuga et al., 2004). In addition, our results showed that LNT stress increased soluble sugar, starch and total nonstructural carbohydrate concentrations in peanut leaves (**Figure 2**). The accumulation of leaf end-products (soluble sugars, starch) is important for balancing photosynthesis and the use of sugars for growth. In particular, there is a close two-way relationship between photosynthesis and growth, rather than a simple dependence of growth on photosynthesis. Carbohydrate synthesis occurs in photosynthetically active leaves (sources); sugars are then exported to support sinks (e.g., for leaf expansion, stem and root growth) (Adams et al., 2013; Cohu et al., 2014). Our results suggested that LNT stress directly inhibits peanut growth and source-to-sink sugar transport, and induces the accumulation of nonstructural carbohydrates in leaves (**Figure 2**), which is consistent with the previous findings in maize (Adams et al., 2013).

LNT stress significantly reduced photosynthetic activity in this study (**Figure 3**). Photosynthesis is the principal process of capturing light energy to form carbohydrates and is very sensitive to the low-temperature environment (Adams and Demmig-Adams, 2004; Stewart et al., 2016). Particularly, we found that the effects of LNT on peanut photosynthesis might be mainly due to reduced peanut growth and leaf expansion, and the export of nonstructural carbohydrates as we only exposed the peanuts to low-temperature during the nocturnal phase. Compared with the normal temperature control, transpiration and photosynthetic rates decreased in response to LNT stress, while intercellular CO_2_ concentration increased (**Figure 3**) which is consistent with findings in maize (Zhu et al., 2010). Photosynthesis and transpiration are often tightly linked, as they both depend on stomatal conductance (Wong et al., 1979; Gago et al., 2016). Based on our results (**Figure 3**), LNT stress had a negative impact on photosynthesis in peanut leaves due to non-stomatal limitations, because C_i_ increased and Ls decreased. The results from the present study are consistent with previous studies on tomato and coffee tree (Bauer et al., 1985; Allen et al., 2000). The non-stomatal limitation was reported previously and attributed to a reduced rate of RuBP regeneration. There are three plausible reasons when RuBP regeneration becomes limiting under low-temperature environment: (i) limitation of the rate at which light-harvesting and electron transport produce ATP and NADPH; (ii) limitation of the rate at which the stromal bisphophatases regenerate RuBP in the photosynthetic carbon reduction cycle; or (iii) restriction of the rate at which end-product synthesis consumes triose-phosphates and regenerates inorganic phosphate (Pi) for photophosphorylation (Allen et al., 2000; Allen and Ort, 2001; Usuda et al., 2006). Taken together, the non-stomatal limitation might be the dominant factor contributing to the down-regulation of photosynthesis under LNT stress.

Our study showed that LNT stress decreased Fv/Fm in peanut, based on chlorophyll fluorescence signal as subtle reflections of the primary reactions of photosynthesis (**Figure 4**). Other studies also suggest that LNT stress followed by warm sunny days with high light can induce severe photoinhibition in tomato and grapevine (Bertamini et al., 2006; Liu et al., 2012). In addition, chilling stress causes irreversible photoinhibition in leaves of other chilling-sensitive plants such as chickpea and cucumber (Sonoike, 1999; Turan and Ekmekçi, 2011). In particular, we found that the effects of LNT on peanut photosynthesis were mediated through sink feedback thereby down-regulating photosynthesis, but induced significant photoinhibition in peanuts leaves. Our interpretation was consistent with previous findings that insufficient sink activity and growth inhibition can lead to severe accumulation of foliar carbohydrates and leading to photoinhibition (Adams et al., 2013). Indeed, the low temperature can inhibit the activities of photosynthetic reaction centers, thus restricting the electron transport chain and carbon fixation rate (Kasuga et al., 2004; Baker, 2008). Our results also showed that thylakoid membranes integrity and ATPase activity decreased during LNT stress (**Figure 7**). LNT stress indirectly increased Y(NO)—the non-regulated energy loss in PSII—indicating the fraction of energy that was dissipated as heat and fluorescence; a high value of Y(NO) reflects the inability of the plant to protect itself against damage by excess excitation (**Figure 5**). It is also plausible that the PSII super-complexes were photodamaged during LNT stress. Both PSI and PSII are sensitive to excess light under chilling stress; PSII is easily inactivated by an excess of excitations and PSI more prone to potential photo-damage caused by excess electrons coming from PSII (Sonoike, 1996; Suorsa et al., 2012). Impairment of the photosynthetic electron transport chain from the donor side of PSII and up to the reduction of end-acceptors of PSI likely limits the production of reduction equivalents and alters the rate of carbon fixation (Song et al., 2018). In this study, the decline in linear electron transport and increase in cyclic electron transport lead to an increase in P700^+^, thereby increasing Y(ND) accordingly (**Figures 5** and **6**). In addition, the low-temperature condition restricts the Calvin cycle, reducing the need for NADPH (Huang et al., 2012). In due course, NADPH accumulates downstream of PSI, altering the ATP/NADPH ratio and causing an over-reduction of the PSI acceptor side (Müller et al., 2001; Rumeau et al., 2007; Noctor et al., 2014).

### Ca^2+^ Improved Peanut Growth and Photosynthetic Capacity During Low Nocturnal Temperature And Its Recovery

Our results showed that foliar application of Ca^2+^ enhanced leaf growth and dry matter accumulation of peanut roots, stems and leaves under LNT stress and improved the recovery (**Figure 1**). Previous studies have also shown that the pre-treatment of exogenous Ca^2+^ improves plant growth and photosynthesis, and enhances cold resistance (Brauer et al., 1990); for example, in peanut (Liu et al., 2013), wheat (You et al., 2002), Chinese crab apple (Li et al., 2017) and tomato (Zhang et al., 2014; Liu et al., 2015). High-yielding peanuts are a calcium-demanding oil crop, with calcium critical for peanut growth and development (Wan, 2003). The LNT + Ca treatment restored the concentration of chlorophyll a to a level similar to that in CK, while the concentration of chlorophyll b was lower than that in CK while higher than that in LNT. Peanut seedlings in the LNT + TFP treatment showed more severe growth inhibition and had lower levels of the main photosynthetic pigments (**Table 1**). Our results also indicated that exogenous Ca^2+^ relieved the excess accumulation of nonstructural carbohydrates (fructose, glucose, sucrose and starch) in peanut leaves under LNT stress (**Figure 2**). It is a well-established principle that plant growth and carbohydrate metabolism are closely linked since carbohydrates generated by photosynthesis are the primary source of building blocks and energy for the production and maintenance of biomass (Osorio et al., 2014). In particular, Ca^2+^, involved in regulating carbohydrate metabolism of plants, can contribute to the regulation of sucrose synthesis, such as the inhibition of cytosolic Fru1, 6bisPase and the activation of SPS (Sucrose-Phosphate Synthase) as well as the turnover of PPi (Brauer et al., 1990; Eckardt, 2001; Lu et al., 2013). In addition, Ca^2+^ is an important component of several signal transduction pathways including sugar signaling (Ohto and Nakamura, 1995; Gounaris, 2001), and Ca^2+^ regulation has been implicated in phloem function (Eckardt, 2001). Furthermore, our results showed that supplementary Ca^2+^ indirectly ameliorated the decline of g_s_ and Tr and maintained C_i_ during LNT which prevented a major decline in Pn (**Figure 3**), which is in accordance with previous studies on *Arabidopsis* (Dong et al., 2013), cotton, tomato, and spinach (Joham, 1957; Brauer et al., 1990), where calcium improved the synthesis, phloem loading and translocation of photosynthetic carbohydrates (Joham, 1957; Eckardt, 2001; Lu et al., 2013). Taken together, exogenous Ca^2+^ application alleviated temperature-dependent photosynthesis feedback inhibition due to improved growth demand and reduced accumulation of nonstructural carbohydrates.

Exogenous Ca^2+^ can relieve photodamage as well as accelerating photosynthetic recovery in peanut leaves under LNT stress. The current study demonstrated that exogenous Ca^2+^ enhanced the PSII self-repairing process under LNT stress and during its recovery (**Figures 4** and **5**). Previous research has shown that the calcium-binding protein CAS is crucial for maintaining PSII activity, recovery, and turnover, as well as for driving high-light acclimation (Terashima et al., 2012). PSII is a multisubunit protein-pigment complex containing polypeptides, both intrinsic and extrinsic to the photosynthetic membrane. The extrinsic luminal protein PsbO can bind to calcium ions and stabilize the function of the oxygen-evolving complex (Heredia and Rivas, 2003; Sasi et al., 2018). In our study, exogenous Ca^2+^ decreased Y(NO) under LNT stress, whereas LNT and LNT + TFP increased Y(NO) indirectly (**Figure 5**). This suggests that the PSII reaction centers under the LNT and LNT + TFP treatments experienced severe photodamage. Conversely, the thylakoid lumen acidification (**Figure 7**) driven by CEF (**Figure 6**) with exogenous Ca^2+^ pretreatment under LNT stress possibly promoted calcium binding to PsbO, which is important in the assembly and stabilization of PSII reaction center (Yi et al., 2005). Moreover, Ca^2+^ might affect the expression of LHC stress-related protein 3, which is crucial for qE, the energy-dependent component of NPQ (Terashima et al., 2012). However, CEF contributes to the pH gradient across the thylakoid membrane which is required for efficient qE. Foliar application of exogenous Ca^2+^ may also increase the binding of calmodulin to NADK2, which is known to modulate the NAD/NADP balance (Rocha and Vothknecht, 2012). Furthermore, our study showed that ATPase activity was promoted by exogenous Ca^2+^ under LNT stress (**Figure 7**). This finding is in accordance with previous studies on tomato under LNT stress (Zhang et al., 2014) and tobacco under high-temperature stress (Tan et al., 2011). Exogenous Ca^2+^ can also enhance the activities of several key enzymes in the Calvin cycle, which in turn boost cyclic electron transport and PSII reaction center activity (Terashima et al., 2012; Hochmal et al., 2015). Therefore, supplementary Ca^2+^ could indirectly reduce over-reduction damage on the PSI acceptor side of peanut leaves. The LNT + Ca treatment induced a rapid increase of CEF to minimize PSI photodamage (**Figures 5** and **6**). Therefore, exogenous Ca^2+^ could restore both PSII and PSI photodamage as well as accelerating the photosynthetic recovery in peanut leaves under LNT stress.

Our data showed that foliar application of a calmodulin inhibitor (trifluoperazine, TFP) exacerbated the inhibition of growth, dry matter accumulation and photosynthetic gas exchange in peanuts under LNT stress, with poor performance during the recovery stage (**Figures 1**–**5**, **Table 1**). The LNT + TFP treatment increased soluble sugar, starch and total nonstructural carbohydrate concentrations more than LNT (**Figure 2**). We found that LNT + TFP strongly reduced peanut photosynthetic capacity through its limitation on peanut growth, leaf expansion and nonstructural- carbohydrate export from leaves to support sink growth and development. TFP can enter plant cells through the cell membrane and prevent the formation of a Ca^2+^–CaM complex, thus inhibiting Ca^2+^–CaM effects (Hepler, 2005; Liu et al., 2013). It is plausible that the Ca^2+^–CaM complex plays an important role in facilitating Ca^2+^ signal transduction to restore peanut growth and photosynthetic capacity under LNT stress. The specific molecular mechanism underpinning the Ca^2+^–CaM complex–LNT stress interaction remains to be examined.

## Conclusions

LNT decreased peanut growth and photosynthetic activity. The protective effects of foliar-applied calcium on peanut were mainly due to improved peanut growth and leaf expansion, and the export of nonstructural carbohydrates, secondarily increasing photochemical activity during exposure to LNT and its subsequent warm recovery. Therefore, exogenous Ca^2+^ restored temperature-dependent photosynthesis feedback inhibition by improving sink demand in peanut under LNT stress. In addition, TFP-treated peanut seedlings performed worst during LNT, which further confirmed the protective role of Ca^2+^ in LNT tolerance of peanut.

## Data Availability Statement

All datasets generated for this study are included in the article/supplementary material.

## Author Contributions

YL, TL, XH, and QBS designed the experiment. QBS, QWS, CB, DW, and XH conducted the experiment and collected data for preliminary analysis. YL, JP, CG, and YC further analyzed the data and prepared the manuscript. All authors reviewed and commented on the manuscript. HL, JP, JY, and KS revised the manuscript.

## Funding

This research was funded by the Natural Science Foundation of China (project no. 31772391, 31301842, 31601627), the National Key Research and Development Plan (project no. 2018YFD0201206), the National Peanut Research System (project no. CARS-13- Nutrient Management).

## Conflict of Interest

The authors declare that the research was conducted in the absence of any commercial or financial relationships that could be construed as a potential conflict of interest.

## Abbreviations

AL, Actinic light; ATP, Adenosine triphosphate; C_a_, Atmospheric CO_2_ concentration; C_i_, Intercellular CO_2_ concentration; CaM, Calmodulin; CEF, Cyclic electron flow; DoT, Days of LNT treatment; DoR, Days of normal temperature recovery; ETR(I), Relative electron transport rate in Photosystem I; ETR(II), Relative electron transport rate in Photosystem II; F, Fluorescence yield measured briefly before application of a saturation pulse; Fo, Minimal fluorescence yield of the dark-adapted sample with all PSII centers open; Fo’, Minimal fluorescence yield of the illuminated sample with all PSII centers open; Fm, Maximal fluorescence yield of the dark-adapted sample with all PSII centers closed; Fm’, Maximal fluorescence yield of the illuminated sample with all PSII centers closed; Fv/F, Maximal photochemistry efficiency in Photosystem II; FH1, Fenghua 1; g_s_, Stomatal conductance; H, Plant height; LA, Leaf area; LNT, Low nocturnal temperature; Ls, Leaf stomatal limitation; NAD, Nicotinamide adenine dinucleotide; NADK, Nicotinamide adenine dinucleotide kinase; NADP, Oxidation form of nicotinamide adenine dinucleotide phosphate; NADPH, Nicotinamide adenine dinucleotide phosphate; NPQ, Non-photochemical quenching; PAR, Photosynthetically active radiation measured in μmol quanta m^–2^ s^–1^; Pm, Maximal P700 signal’; Pm’, Real-time P700 signal under light’; Pn, Net photosynthetic rate’; PPF, Photosynthetic photon flux density’; P_red_, P700 reduction coefficient under light’; PSI, Photosystem I; PSII, Photosystem II; qE, The energy-dependent component of NPQ; RH, Relative humidity; TFP, Trifluoperazine; Tr, Transpiration rate; WUE, Water-use efficiency; Y(II) = ΦPSII, Actual quantum yield in PSII under light; Y(NO), Non-regulatory quantum yield in PSII under light; Y(NPQ), Regulatory quantum yield in PSII under light; Y(I) = ΦPSI, Actual quantum yield in PSI under light; Y(ND), Quantum yield of PSI non-photochemical energy dissipation due to donor-side limitation; Y(NA), Quantum yield of PSI non-photochemical energy dissipation due to acceptor-side limitation; △pH, Proton gradient; △ψ, Membrane potential.
